# Pregabalin Toxicity-Induced Posterior Reversible Encephalopathy Syndrome

**DOI:** 10.7759/cureus.25656

**Published:** 2022-06-04

**Authors:** Feng Hsiao, Abraham Ma, Purushothaman Muthukanagaraj

**Affiliations:** 1 Psychiatry, State University of New York Upstate Medical University, Syracuse, USA; 2 Psychiatry, State University of New York Upstate Medical University, Binghamton, USA; 3 Psychiatry, United Health Services, Binghamton, USA

**Keywords:** prescription drug addiction, altered mental state, high-dose methadone maintenance therapy, pregabalin toxicity, posterior reversible encephalopathy syndrome (pres)

## Abstract

Pregabalin, a prescription medication typically used for neuropathic pain, has increasingly been overused and abused. We present a unique case of a 51-year-old woman with a history of polysubstance use disorder and on methadone therapy who presented to the emergency department with altered mental status after consuming an unknown supratherapeutic amount of pregabalin. She was stabilized and discharged. Within 24 hours, she ingested another 1000mg of pregabalin, presenting again with altered mental status, along with tachycardia and hypertension. Computed tomography (CT) without contrast and fluid-attenuated inversion recovery magnetic resonance imaging (FLAIR MRI) revealed subcortical white matter edema in the bilateral frontal and occipital lobes as well as the left parietal lobe, suggestive of posterior reversible encephalopathy syndrome (PRES). The patient recovered after four days of supportive treatment with an antihypertensive and an antiepileptic. PRES is a neurological phenomenon in which vasogenic edema, most commonly accumulating in the posterior parieto-occipital white matter, causes headaches, altered mental status, and seizures. To our knowledge, there has not been an established link between pregabalin toxicity and PRES, and the awareness of this potential complication can help in the early diagnosis and management of the disease to prevent further progression.

## Introduction

Pregabalin is a prescription medication typically used for the treatment of various conditions such as fibromyalgia and neuropathic pain, and historically has been perceived to have a low risk for abuse [1,2]. However, there is escalating concern that pregabalin may be abused for euphoria, potentiation of methadone, altered consciousness state, or to self-medicate for undertreated or undiagnosed medical conditions [1-3]. In this context, we present the first known case of posterior reversible encephalopathy syndrome (PRES) after pregabalin abuse.

PRES is a neurological phenomenon first described by Hinchey et al. in 1996 [4]. Clinically, patients diagnosed with PRES may experience headaches, nausea, vomiting, visual disturbance, seizures, focal neural deficit, cognitive deficit, and alteration in consciousness [5,6]. It has been most studied in the context of chemotherapeutic agents, immunosuppressive or cytotoxic treatments, preeclampsia and eclampsia, infection and sepsis, and autoimmune diseases. While the underlying pathophysiology is not completely clear yet, PRES is characterized primarily by magnetic resonance imaging (MRI) findings of subcortical vasogenic edema, most commonly exhibited as bilateral parieto-occipital and posterior frontal lobe hyperintensities on fluid-attenuated inversion recovery (FLAIR) sequence [5-8]. Treatment involves correcting known underlying causes and managing complications such as seizures. Although most patients recover, PRES is not always completely reversible [9]. Early recognition and intervention can help to reduce morbidity and mortality.

## Case presentation

**Figure 1:**
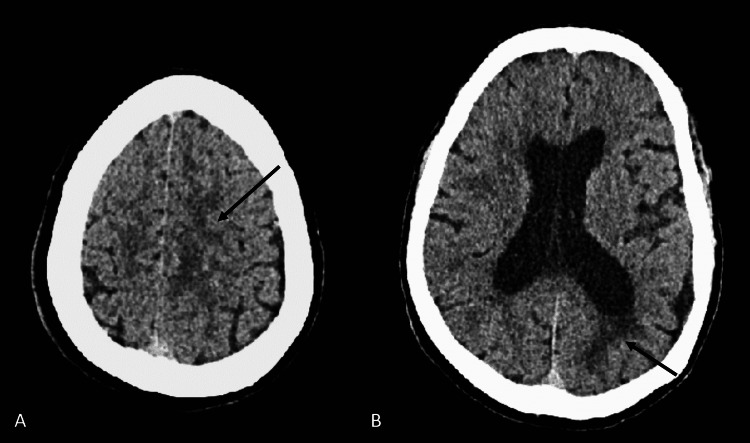
Computed tomography scan of the brain without contrast (A and B) Axial images showing mild to moderate hypodensities in left frontal and occipital lobes, suggestive of white matter disease (arrows).

A 51-year-old woman with a history of schizoaffective disorder, polysubstance use disorder, and HIV on antiretroviral therapy presented to the emergency department with altered mental status. She was being treated daily with 135 mg of methadone, which had been increased from 125 mg recently. She also had a history of multiple overdoses on benzodiazepines and other prescription drugs. Upon presentation, the patient was obtunded and unresponsive to sternal rub, and initial vitals showed a temperature of 36.6°C, blood pressure of 101/75, pulse of 50, respiratory rate of 12, and oxygen saturation at 89% on room air. Selected laboratory test results highlighting the abnormal components are summarized in Table 1. Computed tomography (CT) of the head without contrast showed no evidence of intracranial hemorrhage or infarction, with mild to moderate hypodensities in the left frontal and occipital lobes (Figure 1). Ultrasound and CT of the abdomen and pelvis were unremarkable. Naloxone 0.4 mg injection was given twice to reverse suspected opioid overdose, and she subsequently became agitated, requiring multiple doses of lorazepam 2 mg and haloperidol 5 mg injections over the next 48 hours before becoming more alert and oriented. Afterward, she admitted to abusing and unintentionally overdosing on pregabalin, which was prescribed to her for chronic back pain, and she stated that taking five to six 200 mg tablets gave her a "high" lasting more than one day. Subsequently, she was diagnosed with altered mental status secondary to pregabalin overdose in the background of high-dose methadone maintenance therapy. Once her symptoms resolved and she was deemed stable two days after admission, she was discharged home with recommendations to follow up with her primary care provider to taper down her methadone and pregabalin dosages.

**Table 1:** Summary of the patient's abnormal laboratory test results CD = Cluster of differentiation

Component	Result	Reference range and units
Potassium (K+)	3.2	3.5-5.3 mmol/L
Bicarbonate (HCO3-)	20	21-32 mmol/L
Amylase	340	30-110 U/L
Lipase	2,914	<300 U/L
Creatine kinase	2,152	26-192 U/L
CD4 T cell count	16	34-56 %
CD4/CD8 ratio	0.26	1.00-4.00

**Figure 2:**
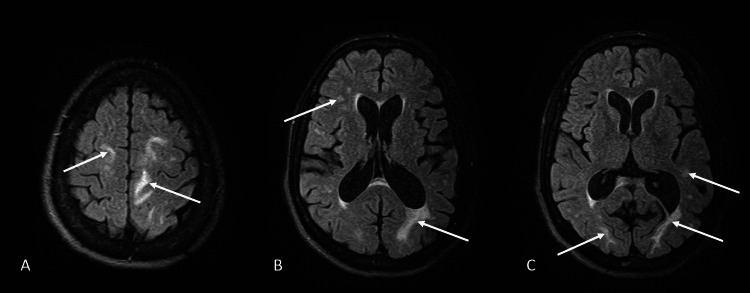
Magnetic resonance imaging of the brain in a patient with pregabalin-induced PRES (A) Axial fluid-attenuated inversion recovery (FLAIR) image showing hyperintensities in bilateral frontal lobes (arrows). (B and C) Axial FLAIR images showing hyperintensities in the right frontal lobe, left parietal lobe, and bilateral occipital lobes (arrows). PRES = posterior reversible encephalopathy syndrome

Within 24 hours after the discharge, the patient presented again with acute worsening of altered mental status, unable to state her birthday nor the current month, year, or place. She was tachycardic with a pulse of 120 and hypertensive with a blood pressure of 166/100. Urine drug screen was negative except for methadone. MRI study of the head without contrast was done, revealing subcortical white matter edema in the bilateral frontal lobes, bilateral occipital lobes, and left parietal lobe (Figure 2). Electroencephalography was performed, which showed a normal study without epileptiform discharges. Progressive multifocal leukoencephalopathy was deemed unlikely because CD4 T cell count was well above 200 cells/uL. Because of hypertension and MRI findings, PRES was diagnosed. Levetiracetam 500 mg twice daily was started due to concerns for possible seizures, and blood pressure control was achieved with amlodipine 10 mg once daily. Over the next four days, she became more oriented and admitted to abusing pregabalin again after the previous discharge, taking five 200 mg tablets. After symptom stabilization, she agreed to voluntary psychiatric admission to better manage her conditions; however, she ultimately decided to leave against medical advice.

## Discussion

Pregabalin is a Schedule V drug approved for the management of fibromyalgia, neuropathic pain associated with postherpetic neuralgia, diabetic neuropathy, and spinal cord injury, as well as adjunct therapy for partial-onset seizures. Increasing reports of pregabalin abuse have been documented, with users stating that the drug can produce euphoric effects when taken in supratherapeutic doses [1,2,10]. The drug is also commonly taken as a co-ingestant with opioids and benzodiazepines among patients with substance use disorder due to its ability to potentiate their effects, and there is emerging evidence that pregabalin is being abused alongside methadone [3,11].

There have been multiple cases documented of acute toxicity of pregabalin causing neuropsychiatric symptoms [11-14]. One case reported an elderly patient with a history of hypertension and diabetes nephropathy, who accidentally overdosed on pregabalin and presented with altered mental status secondary to reversible toxic encephalopathy manifesting as continuous triphasic waves in EEG [12]. In a similar case, a patient treated with pregabalin for years became mentally altered with an EEG revealing continuous triphasic waves after developing prerenal azotemia [13]. In both cases, impaired renal clearance of pregabalin was the likely cause of intoxication, and the patients had a complete recovery of mental status following the discontinuation of the offending drug.

Although there is no known documentation of PRES precipitated by acute pregabalin toxicity, a few reports of rare cases have shown that PRES can occur with certain neuromodulating medications, including duloxetine and gabapentin [15,16]. Notably, pregabalin and gabapentin both belong to the gabapentinoid drug class and therefore share similar pharmacodynamic profiles. However, pregabalin is 2.5 times more potent with quicker absorption and higher bioavailability than gabapentin, so it is reasonable to assume that pregabalin has higher abuse potential and can also cause PRES [2].

Recently, a patient reportedly presented with a three-month history of behavioral changes and abnormal movements, and MRI showed subtle FLAIR hyperintensities in the right cerebellum, right cingulate gyrus, and left posterior limb of the internal capsule [14]. Due to the patient’s history of psoriasis and given that his symptoms responded well to methylprednisolone, the authors initially diagnosed him with autoimmune encephalitis. However, after his discharge, he experienced an acute worsening of symptoms and presented to the hospital again, at which point he admitted that he had been taking 2,250 mg of pregabalin daily for two years. The patient was never diagnosed with PRES, and the locations of his MRI findings would be an atypical pattern for the disease. Nonetheless, the similarities between this case and our patient raise suspicion that the underlying causes of their clinical pictures might share a similar mechanism.

The exact mechanism of PRES is still unclear. One hypothesis states that hypertension leads to failed cerebral auto-regulation, resulting in hyperperfusion, endothelial damage, and breakthrough vasogenic edema; however, hypertension is not present in all PRES cases [6,8]. Another hypothesis is that endothelial dysfunction, brought on by various triggers such as autoimmune diseases, infection, and chemotherapeutic agents, affects the integrity of the blood-brain barrier and therefore causes vasogenic edema [7]. Common drugs of abuse, such as opioids and cocaine, are noted to compromise the blood-brain barrier, which may explain the reports of opioids, including methadone, causing PRES [17-20].

Our patient had been on a relatively high dose of chronic methadone maintenance therapy and was found to be a habitual abuser of pregabalin. Although her clinical symptoms and MRI findings suggestive of PRES were induced by an acute overdose of pregabalin, it is hard to discern whether it could have been caused by the drug’s own pharmacological action or by its potentiating methadone’s effect. Therefore, further research is warranted to elucidate the mechanistic connection between pregabalin and PRES. Nevertheless, this case is evidence that a prescription of pregabalin for patients on methadone should be exercised with caution. Although most patients with PRES recover, it is not always reversible, as the lesions can progress to permanent local ischemia and leukomalacia if left untreated [9]. Therefore, greater emphasis needs to be placed on identifying risk factors and monitoring for signs of pregabalin abuse to reduce morbidity and mortality among patients with a history of substance use disorder.

## Conclusions

PRES is typically associated with preeclampsia, chemotherapy, and immunosuppressive agents but has been reported to occur with other medical problems and medications. To our knowledge, we present the first reported case of PRES precipitated by pregabalin. Awareness of this potential association is important since pregabalin abuse has become an increasingly known entity, and early recognition and treatment of PRES may reduce the risk of permanent neurological deficits. PRES should be considered as a differential diagnosis in patients with a history of substance abuse and pregabalin treatment that present with hypertension and neurological symptoms.
